# Treatment of Typhoid Fever in Paris

**Published:** 1853-07

**Authors:** 


### RECORD OF MEDICAL SCIENCE.

### PATHOLOGY AND PRACTICE OF MEDICINE.

*Treatment of Typhoid Fever in Paris.*—An epidemic of Typhoid Fever is very prevalent in Paris at present, and it is seen in all the Hospitals. In these it is quite interesting to witness the variety of treatment adopted by each physician of note, as his medical tendencies incline; that pursued respectively by MM. Bouillaud, Briquet and Louis, whom I have followed, presents many points of difference, and it has been difficult to determine on which side the scale of success leans. We hope, however, to be able to send a paper by a medical friend who has watched closely the service of the former, and who can furnish some testimony with respect to his mode of employing the lancet in this disease. M. Louis very generally administers seltzer water, vinous lemonade, and adopts, to a certain extent, the expectant system. M. Briquet at La Charite pursues to me rather a middle course. He abstracts blood in the early stage of some cases which seem peculiarly fitted for it. I may observe here that I also have heard M. Trousseau say, that even as late as during the second and third septenary period, he was in the habit of bleeding to prevent the increase, or destroy the congestion of the lungs which so often exhibits itself, its approach and presence being of course easily discoverable by the physical signs. M. Briquet relies with much confidence (and my own observation in his wards can sustain him) upon the application of cups and scarification to the mastoid region during delirium and coma of Typhoid. It completely checks the increase of these dangerous symptoms, as I have repeatedly witnessed. He does not give mercury internally, but applies plasters of mercurial ointment over the entire abdomen until the secretions become active, or the tongue and skin exhibits the desired moisture. This method of using the remedy might more easily reconcile itself to those who so much doubt respecting the advantages of its internal administration. He gives ordinary Bordeaux wine, during the period of convalescence. These means, thus generally expressed, are often associated in his practice with the use of ice, administered in every possible way, and through every channel—bladders filled with it are applied to the scalp, it is dissolved in the mouth and given with enemata. One of his Internes mentioned to me this morning, that he had lost but one case within three weeks. There are between twenty and fifty sick of the disease in each service of the Hospitals, and few of these fail to present gargouillement, the character rose mark and sudamina.—*Paris Correspondence Charleston Med. Journ.*, June, 1853.

